# Saving coral reefs: significance and biotechnological approaches for coral conservation

**DOI:** 10.1007/s44307-024-00049-1

**Published:** 2024-11-22

**Authors:** Pansa Cecchini, Thomas Nitta, Edoardo Sena, Zhi-Yan Du

**Affiliations:** Department of Molecular Biosciences & Bioengineering, University of Hawaiʻi at Mānoa, Honolulu, HI 96822 USA

**Keywords:** Coral, Coral bleaching, Conservation, Climate change, Resilience, Symbiosis, Zooxanthellae

## Abstract

Coral reefs are highly productive ecosystems that provide valuable services to coastal communities worldwide. However, both local and global anthropogenic stressors, threaten the coral-algal symbiosis that enables reef formation. This breakdown of the symbiotic relationship, known as bleaching, is often triggered by cumulative cell damage. UV and heat stress are commonly implicated in bleaching, but other anthropogenic factors may also play a role. To address coral loss, active restoration is already underway in many critical regions. Additionally, coral researchers are exploring assisted evolution methods for greater coral resilience to projected climate change. This review provides an overview of the symbiotic relationship, the mechanisms underlying coral bleaching in response to stressors, and the strategies being pursued to address coral loss. Despite the necessity of ongoing research in all aspects of this field, action on global climate change remains crucial for the long-term survival of coral reefs.

## Introduction

Coral reefs are complex ecosystems that are found in oceans worldwide and make up one of the most productive environments on Earth (Brandl et al. 2019; Hoegh-Guldberg et al. 2017; Munday et al., 2008). Corals are members of the class Anthozoa, consisting of hard corals, soft corals, and sea anemones. Hard corals are members of the order *Scleractinia* and build large structures through the excretion of calcium carbonate skeletons. These reefs not only provide ecosystem services for marine life but also hold economic, cultural, and natural services, with the value of these processes placed at $20 trillion US dollars annually (Roth 2014). Human impact in the Anthropocene has caused a significant decline in reef health and cover, and management is crucial in order to sustain the economies of coastal countries and the millions of residents who depend on reefs for various purposes (Brandl et al. 2019; Grafeld et al. 2017; Moberg & Folke 1999; Woodhead et al. 2019).

Coral reefs of the tropics host immense biodiversity, which stems not from the coral species themselves (estimates of 835 reef-building coral species), but rather from the 1–9 million species of organisms that reside in coral reefs (Knowlton 2001; Reaka-Kudla et al. 1997). Coral reef cover of the ocean floor ranges from 0.1% to 0.5%, yet they are estimated to hold 25% to 33% of all marine species, including fishes, corals, marine invertebrates, and plants (Knowlton et al. 2010; Moberg & Folke 1999; Sebens 1994). The biodiversity of reefs has been studied extensively, and results have indicated a positive relationship between biodiversity and ecosystem productivity, nutrient cycling, and overall enhanced ecosystem function (Brandl et al. 2019; Gamfeldt et al. 2015). This observation is dubbed a “biodiversity-ecosystem functioning” relationship, but the specific causations of this relationship in coral reefs and how coral and coral-dwelling organism diversity influence reef productivity is an emerging field of study (Hooper et al. 2012; McWilliam et al. 2018). It is known, however, that a healthy, functioning reef ecosystem is essential for both direct and indirect benefits to the lifestyles of millions of people, particularly those living in coastal locations near coral reefs (Woodhead et al. 2019). Additionally, having a rich diversity of species is vital for the scientific field, as the reefs contain important “genetic libraries” that could provide services such as assessments of reef health (Moberg & Folke 1999; Woodhead et al. 2019).

One of the driving factors that allows for such high biodiversities of organisms from a wide taxonomic range is the complex topography created by hard reef-building corals themselves. This structural complexity creates many microhabitats for various species and provides many organisms with shelter, food, and novel niches for organisms to exploit (Graham & Nash 2013; Rogers et al., 2014). There have been various studies that have shown positive associations between the structural complexity of the environment and species biodiversity not only in coral reefs (Graham & Nash 2013; Gratwicke & Speight 2005; Luckhurst & Luckhurst 1978) but also in ecosystems such as terrestrial forests or seagrass beds (Heck & Wetstone 1977; Spies 1998). All species in an ecosystem play a specific functional role, and high biodiversity allows for these functions to continue even after a decline of certain species. (Wilson et al. 2009). The leading causes of decreases in biodiversity are habitat loss and degradation. As crucial foundation species, healthy corals are necessary to maintain coastal reef habitats with high biodiversity.(Hoekstra et al. 2005; Pratchett et al. 2014).

Across all marine ecosystems, fishes are often the most well-documented organisms as they are high in abundance and play important roles ecologically and economically (Cole et al. 2008; De Mitcheson & Erisman 2012; Duffy et al. 2016; Hixon 2011; Mora et al. 2008). It is very difficult to accurately define the global abundance of reef fishes, with some estimates of up to 8000 unique species (Hixon 2011). Many different fish taxa serve unique, important ecological roles, including corallivorous, herbivorous, carnivorous, and planktivorous fishes (Cole et al. 2008b; Kulbicki et al. 2005; Mantyka & Bellwood, 2007; Ogden & Lobel 1978; Siqueira et al. 2021). Herbivorous fishes are examples of reef fish that indirectly contribute to reef health through the consumption of macroalgae, which can outcompete corals for space on a reef and have an even larger impact on reefs in which coral cover is declining (Hughes et al. 2007; Plass-Johnson et al. 2015). Fishes of different trophic levels may not contribute directly or indirectly to coral health. However, all play a role in maintaining biodiversity through intra- and interspecific interactions, such as through competition or predation (Boaden & Kingsford 2015; Boström-Einarsson et al. 2013; Robertson 1998). Complex topography has been correlated with higher abundances of adult fishes (Graham & Nash 2013; Gratwicke & Speight 2005), facilitates larval settlement, and provides shelter for juveniles (up to 65% of fishes may require coral cover for survival at settlement) (Jones et al. 2004; Wilson et al. 2009).

Of course, fish are not the only abundant fauna on coral reefs as there are many invertebrates, both sessile and mobile, that contribute to the biodiversity and health of coral reefs but are less accounted for in literature compared to fishes. This research gap is unfortunate, given that invertebrates account for the greatest biodiversity on reefs (Stella et al. 2011). The lack of literature is largely in part due to the research bias of conspicuous organisms (such as fishes and corals) and the complexity of coral reefs, which creates lots of habitats that are difficult to study, such as those of cryptic invertebrates (Idjadi & Edmunds 2006; Knowlton et al. 2010; Nelson et al., 2016; Stella et al. 2011). However, it is known that certain invertebrates join reef fishes in playing key ecosystem roles, such as herbivorous urchins grazing on macroalgae species, which can prevent significant overgrowth of algae on a reef (Nozawa et al. 2020; Ogden & Lobel 1978). Most urchins are nocturnal and take advantage of the structural complexity of the reef to hide from predators during the day, and at night they emerge to feed (Young & Bellwood, 2011). Other studies have shown overall increases in the biodiversity of invertebrates with more complex topography, which further emphasizes the importance of having high abundances of hard coral (Roth 2014; Yamashita et al., 2014).

The diverse array of organisms present on coral reefs provides important ecosystem services to over 100 countries that contain coral reefs on their coastlines (Moberg & Folke 1999). The economies of these countries directly benefit from reef-based industries, including fisheries, tourism, aquarium trades, and harvesting of natural resources (Cesar & Van Beukering 2004; Grafeld et al., 2017; Moberg & Folke 1999; Spurgeon 1992; Woodhead et al. 2019). Small-scale fisheries in many smaller coastal nations provide food, as well as income through the selling of catches to members of local communities or to larger commercial fisheries (Grafeld et al. 2017; Woodhead et al. 2019). In Hawaiʻi, nearshore fisheries yield over 7 million meals annually, as well as over $10 million USD worth of catch per year (Grafeld et al. 2017). Reef tourism provides another major economic benefit for coastal communities, with an economic value of upwards of $35.8 billion USD annually (Spalding et al. 2017). Smaller nations such as the Maldives also rely more heavily on tourism for economic development compared to other methods (Spurgeon 1992). Lastly, various natural resources can be harvested to support economies, such as obtaining fish or coral for the aquarium trades or using coral to create lime and cement for building infrastructure (Moberg & Folke 1999; Spurgeon 1992). With approximately 7.5% of the global human population dependent on reefs to some extent, maintaining healthy reefs is crucial (Madin & Madin 2015; Speers et al. 2016).

Activities provided by coral reefs are often dependent on the quality of the reefs themselves. Reefs with many attributes such as structural complexity or coral species diversity provide more habitat for marine organisms such as fish or invertebrates, which in turn create more economic profit through local or tourist use (Pascal et al. 2016). Coral reef fisheries are highly dependent on the abundance of these attributes to support populations of species that are fished (Cesar & Van Beukering 2004b; Nadon et al. 2015). Coral reef fishery revenues are valued at approximately $6 billion, and support 6 million fishers across the globe (Hoegh-Guldberg et al. 2019; Nash & Graham, 2016). Across the other countries with populations along coastal reefs, many rely on coral reef fisheries for food and income; there are many fishing households facing poverty that rely heavily on the local fishing industry for their daily sustenance (Albert et al. 2015; Bell et al. 2018; Cabral & Geronimo 2018; Nash & Graham, 2016). Anthropogenic pressure on coral reefs has placed these fisheries in critical states, as the catch per unit effort of reef fisheries has declined by 60% since 1950, following the trend in global coral cover decline (Eddy et al. 2021).

Globally, the total GDP from coral reefs is highly variable; data collected by Cesar et al., 2003 showed that 0.2% of Indonesia’s GDP came from reef-related services, whereas in Bermuda, reef-related services contributed to 12% of the country’s GDP (Allemand & Osborn, 2019). The economic benefit comes from fishing, tourism, and coastal protection due to reef-building corals (Allemand & Osborn, 2019; Chen et al., 2015; Hoegh-Guldberg et al. 2019; Narayan et al. 2016; Pascal et al. 2016; Spalding et al., 2017b). Spalding et al. (2017) projected the global value of tourism to coral reefs to be approximately $35.8 billion. Small island countries, in particular, are heavily reliant on reef-related tourism to support their economy (Hampton & Jeyacheya, 2020). For example, the Republic of Palau, an island archipelago in the North Pacific, has 8% of its GDP supported solely by the tourist attraction of diving with sharks on the reefs of Palau (Vianna et al. 2012).

A passive role coral reefs play to benefit the economy also comes from coastal protection. The strong structures created by hard corals can act as barriers that dissipate incoming wave energy through friction or breaking waves (Beck et al. 2018; Buckley et al. 2022; Symonds et al. 1995). This function protects coastal areas from flooding due to storms and coastal erosion (Cesar and van Beukering 2004; Pascal et al. 2016; Van Zanten et al. 2014). Coral erosion also forms sediment that goes on to comprise the beaches and nearshore habitats of many coastal locations (Brown et al. 2021; Dawson and Smithers 2014; Harney and Fletcher, 2003; Morgan and Kench, 2016). Beck et al. (2018) modeled the value of protection that coral reefs provide coastal communities at over $4 billion annually. Without reefs, the annual damage could more than double that. With sea level rise and the frequency of tropical storms increasing, the loss of reef structure can be detrimental to many coastal countries and their residents (Bhatia et al., 2019; Van Zanten et al. 2014).

In addition to benefits directly to coastlines, coral reefs enhance neighboring marine ecosystems’ services. As an example, seagrass meadows store a large amount of atmospheric carbon dioxide (Duarte et al. 2005; Fourqurean et al. 2012; Mcleod et al. 2011). These ecosystems are facilitated by adjacent coral reefs, which provide natural barriers to the seagrass beds and allow them to better capture organic material (Guerra-Vargas et al. 2020; Ranith et al. 2024). Reef degradation can lead to a parallel loss of carbon storage efficiency in the neighboring seagrass meadows (Guerra-Vargas et al. 2020). Other ecosystems, such as mangroves, similarly benefit from nearby coral reefs (Guannel et al. 2016). Further research on ecosystem interconnectivity could illustrate the reciprocal benefits provided to both coral reefs and their adjacent ecosystems.

On a global scale, coral reefs play integral roles in many important biogeochemical cycles. For example, coral reefs are responsible for a great deal of oceanic carbon dioxide (CO_2_) exchange due to both the calcification of corals themselves and the organic matter flux within them (Gattuso et al. 1995; Kleypas et al. 2011; Lin et al. 2024). Reef interactions with CO_2_ are highly complex. Some studies describe reefs as net sinks and others as sources; many factors, such as seasonality, contribute to the variability (e.g., Bates 2002; Jiang et al. 2011; Kayanne et al., 1995; Suzuki and Kawahata 2003). Though they undoubtably play a role (see Watanabe and Nakamura 2019 for more information), further research is necessary to more accurately assess the contribution of coral reefs to global carbon dynamics. It is worth mentioning that the production of calcium carbonate (CaCO_3_) may contribute to buffering the effects of ocean acidification, indicating that coral reefs influence ocean pH (Kleypas et al. 2011). In addition to carbon, coral reefs influence nitrogen bioavailability in the ocean. N_2_-fixing diazotrophs associated with the stony coral microbiome contribute to the total amount of bioavailable nitrogen in the ocean (Cardini et al., 2014; Pogoreutz et al., 2017). Coral rubble and dead coral are also associated with diazotroph activity, marking the importance of coral structures in general as diazotroph microhabitats (Davey et al. 2008; El-Khaled et al. 2021; Shasar et al.,^ 1994^).

**Fig. 1:**
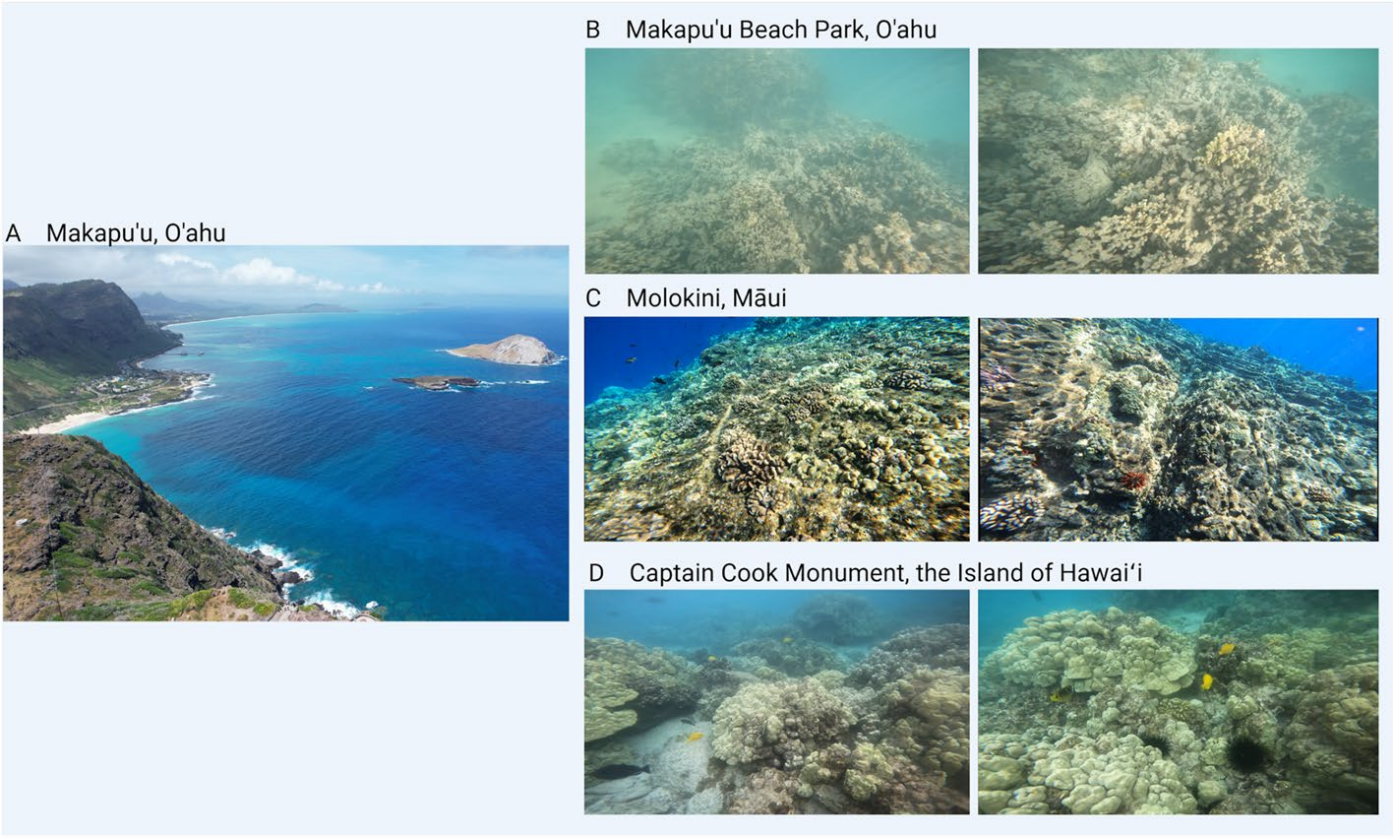
(**A**) Coastal reefs, such as the one shown in the aerial view from Makapu'u, O'ahu, Hawai'i, provide many benefits including tourism, fishery, and coastal protection. (**B**) Serious coral bleaching was observed in photos from coral reefs at the Makapu'u Beach Park, O'ahu. Partially bleached and threatened coral reefs at (**C**) Molokini, Māui and (**D**) Captain Cook Monument, the Island of Hawaiʻi. Photos taken by Z.D. in 2022

Coral reefs have been under anthropogenic threat for decades due to factors including overfishing, pollution, and increasing pressure on coastlines as human populations increase (Ateweberhan et al. 2013; Hughes et al. 2017; Kleypas et al. 2001). The addition of climate change as an ongoing threat has greatly accelerated the rates of species loss in coral reefs (Hooper et al. 2012; Hughes et al. 2017). Climate change has caused two significant stressors to coral reefs—ocean acidification and global warming of seawater (Anthony et al., 2008; Ateweberhan et al. 2013; De’Ath et al. 2012; Kleypas et al. 2001). Coral reefs are facing significant bleaching issues worldwide, with notable examples observed in the Hawaiian Islands (Fig. 1). This review presents how the ecological and economic importance of coral reefs are under threat as the rate of coral reef loss drastically increases due to localized unsustainable human activity and global-scale environmental damage (Anthony et al., 2008; Brandl et al. 2019; Hughes et al. 2017; Moberg & Folke 1999). As this review is considered a primer, it contains a general overview of the coral symbiotic interactions and bleaching response. It further summarizes the threats to corals, particularly heat and UV stress, among other factors. This review also compiles research on restoration and assisted evolution strategies to mitigate coral loss and improve future resilience. The many topics discussed herein are intended to provide an in-depth and current overview of coral science, aid in identifying knowledge gaps, and highlight pathways for future research.

## Symbiotic interactions

**Fig. 2:**
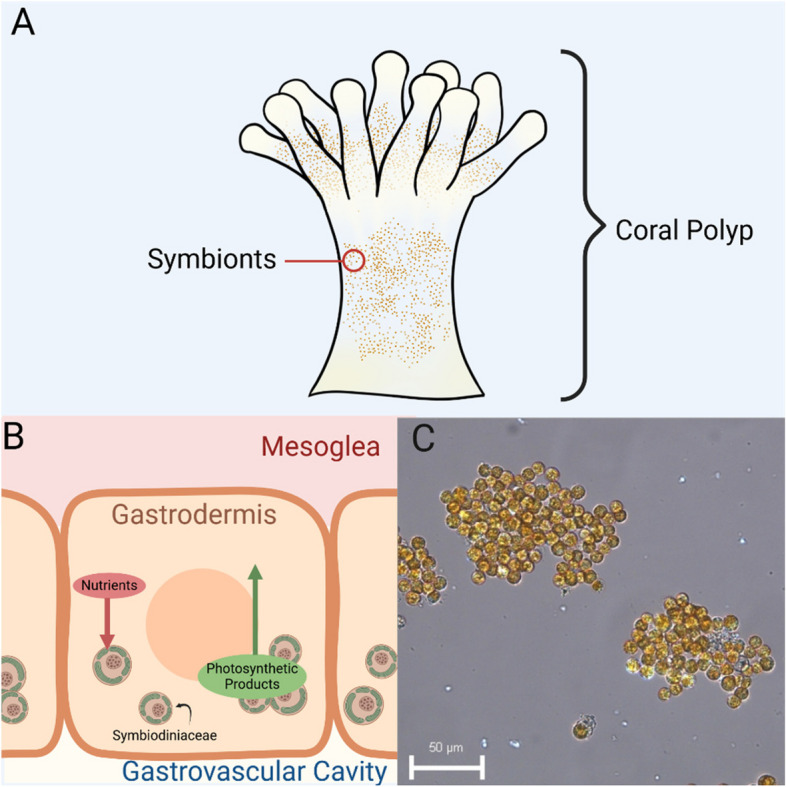
(**A**) Diagram of a coral polyp showing where the algal symbionts reside. (**B**) Simplified schematic of the metabolic relationship of Symbiodiniaceae. They reside in the gastrodermal cells of coral polyps. (**C**) Free-living symbionts of species *Breviolum minutum*. Each cell is roughly 10 μm in diameter

The diversity and size of structures corals can build, and the values these structures provide to hundreds of millions of organisms (including humans) dependent on them are not created by the corals alone. The success of reef-building corals is dependent on their mutualistic symbiotic relationship with the single-celled photosynthetic dinoflagellates within the family Symbiodiniaceae, colloquially called zooxanthellae (Fournier 2013). They reside in the gastrodermal cavity of coral polyps and provide corals with photosynthetic products (organic carbon and amino acids) that can supply over 90% of the coral's carbon needs, allowing for improved coral growth and calcification rates, survival, and reproduction (Fig. 2; Curran & Barnard 2021; Davy et al. 2012; Goreau & Goreau 1959; Roach et al. 2021; Stat et al. 2008; Tambutté 2011; Tanaka et al. 2018) In return, the coral host provides the dinoflagellates with metabolic waste products such as ammonia and phosphate, as well as a fixed position in the water column to allow for a steady supply of light energy (Davy et al. 2012; Hoegh-Guldberg 1999; Roth 2014). Coral hosts may obtain their symbionts through transmission from parent to offspring (vertical transmission; Hirose et al. 2001), or through uptake from the environment (horizontal transmission; Berkelmans & van Oppen 2006; Finney et al. 2010). The specificity of symbionts may vary across regions and with environmental factors such as temperature and nutrient availability (Berkelmans & van Oppen 2006; Finney et al. 2010). This variable, nutrient-recycling symbiosis is critical for the construction of complex habitats that support millions of species in oceans and seas that are typically oligotrophic (Birkeland 1988; Cardini et al. 2015; Stanley Jr. & van de Schootbrugge 2009). In this symbiosis, algal symbionts provide photosynthetic products while the coral hosts provide shelter, access to nutrients derived from the surrounding ocean water, and access to host-derived nutrients such as nitrogenous wastes (Davy et al. 2012; Lewis 1973; Muscatine et al. 1984; Rinkevich 1989). This relationship enhances coral calcification rates and enables the construction of vast reef structures (Goreau & Goreau 1959; Tambutté 2011).

Besides nutrient cycling, symbiotic algae associated with coral hosts gain a direct benefit of improved light absorption efficiency. The light-scattering effect of the coral skeleton greatly enhances the ability of algal symbionts to capture light (Enríquez et al. 2005, 2017; Kramer et al. 2022). This optical scattering has allowed coral-algal symbiosis to thrive even in mesophotic areas with low incident irradiance (Kramer et al. 2022). Enríquez et al. (2017) suggest that the symbiotic coral skeleton morphology evolved specifically to improve algal light harvesting efficiency, thereby increasing the availability of photosynthetic products to the host coral. Furthermore, the coral tissue itself modulates incident light, creating light gradients that form highly complex microhabitats for algae (Wangpraseurt et al., 2012, 2019). These microniches allow the algae to both maximize their photosynthetic efficiency and avoid excess light stress, again leading to greater fitness for the entire holobiont (Wangpraseurt et al. 2012). The optical properties of coral indicate the antiquity and interdependency of the coral-algal symbiosis. Algal symbionts were formerly organized under several clades within the genus *Symbiodinium*. As of 2018, *Symbiodinium* clades have been organized into several genera under the family name Symbiodiniaceae, based on molecular evidence (LaJeunesse et al. 2018). This reorganization elucidates the large degree of variation within the family; genera may differ in thermal tolerance, photosynthetic productivity, and other such properties (Berkelmans & van Oppen 2006; Curran & Barnard 2021). The more resilient of these genera may repopulate the coral host following a bleaching event, thereby achieving thermal adaptation (Bowling 2022; Russnak et al. 2021). Of particular relevance to bleaching is what is now referred to as *Durisdinium* spp. (formerly Clade D), which is known for its high thermal tolerance (Berkelmans & van Oppen 2006; Curran & Barnard 2021). Research is ongoing to better understand the physiological differences between Symbiodiniaceae and their effects on the coral host (Russnak et al. 2021; Swain et al. 2021; Torres et al. 2021). Such research will be further explained later in this review.

The coral-algal symbiosis requires careful regulation by both members (Mayfield & Gates 2007), such as through protein phosphorylation (Simona et al. 2019), transcriptomic regulation (Zhang et al. 2022), host immune responses (Mansfield et al. 2017; Zhang et al. 2022), and other molecular signaling (Rosset et al. 2021). Excessive levels of algal symbionts, poor photosynthetic productivity, and other such factors may elicit a response from either symbiont (Davy et al. 2012; Muscatine et al. 1998; Oakley & Davy 2018). These responses will be further explored throughout this paper. Symbiotic cnidarians have been shown to downregulate immune and inflammatory responses when compared to aposymbiotic counterparts (Lehnert et al. 2014; Wolfowicz et al. 2016), suggesting that cnidarian immune systems must demonstrate tolerance to symbiotic organisms (Mansfield & Gilmore 2019). The immune system may also distinguish between preferred Symbiodiniaceae genera, employing pathways similar to reactive oxygen species (ROS)-related-bleaching in order to remove undesired symbionts (Matthews et al. 2017). The recognition of Symbiodiniaceae, including specific genera, may be explained by pattern recognition receptors, which serve to detect microbes in the host innate immune system (Mansfield & Gilmore 2019). These pattern recognition receptors are also able to detect signs of cellular damage, such as heat shock proteins (Rosenstiel et al. 2009), suggesting a dual role in innate immunity and bleaching (Mansfield & Gilmore 2019). The innate immune response has been induced in bleaching events, as evidenced by the modulation of inflammation and other immune mechanisms in response to environmental stressors and symbiont presence (Mansfield & Gilmore 2019).

The coral microbiome is not limited to symbiotic algae and includes bacteria, viruses, fungi, and archaea (Bourne et al. 2016; van Oppen & Blackall 2019). In particular, bacterial composition has been the subject of much research, and bacterial communities are implicated in affecting coral physiology (Osman et al. 2020; Santoro et al. 2021). Research is ongoing to better understand the interactions between members of the coral microbiome, such as the effects of ocean acidification, bacterial infection on microbial community compositions, and the interplay between bleaching and microbiome (Barreto et al. 2021; Santoro et al. 2021; Sun et al. 2023). Microbiome interactions are beyond the scope of this review, but it is worth mentioning for its growing contribution to the coral research field. Additional information on the coral microbiome and its influence on the holobiont have been well-discussed in other papers (see Mohamed et al. 2023; van Oppen & Blackall 2019). Continuing research on the microbiome beyond algae is important to fully understand the complexity of symbiotic interactions that confer host fitness. Of particular interest is multi-omics data that could help inform microbe-host interactions, including both metabolic and immune functions, among others (Mannochio-Russo et al. 2023; Pogoreutz et al. 2022; Williams et al. 2021).

The coral-algae symbiosis is hypothesized to have persisted since the Triassic period, but during the last half-century, this symbiosis has been placed under extreme stress (Lough et al. 2018; Schoepf et al. 2015; Stanley Jr. & van de Schootbrugge 2009). Environmental pressures that stem from anthropogenic activity affect corals globally (global warming, ocean acidification) or locally (pollution, overfishing, coastal development, and over-exploitation of the ecosystems) (Douglas 2003; Hoegh-Guldberg 2011; Hughes, Kerry, et al. 2017; Roach et al. 2021). Coral “bleaching” is a term used to describe the state of corals once they lose their symbiotic dinoflagellates or the pigments of the algae degrade, leaving the tissues translucent (Douglas 2003; Plass-Johnson et al. 2015). Bleaching renders the coral colony white, the color of their calcium carbonate skeleton (Ainsworth & Brown 2021; Brown 1997; Douglas 2003; Hoegh-Guldberg 1999). Once the symbionts are lost, if they are not recovered within weeks to months (this requires external conditions to return to normal), corals are threatened with mortality due to lack of food, damage to cells, and loss of energy reserves; on a large scale, this can threaten the health of an entire reef (Ainsworth & Brown 2021; Ainsworth & Gates 2016; Douglas 2003).

The earliest documented widespread coral bleaching events were in 1983 during an intense El Niño event, and the first global mass coral bleaching event was during the next extremely strong El Niño in 1998 (Coffroth et al., 1990; Eakin et al. 2019). Since these first modern bleaching events, databases have estimated 22,650 positive bleaching reports, and possibly up to 71% of the world’s coral reefs have experienced bleaching at least once from 1985 to 2017 (Virgen-Urcelay & Donner, 2023). At the time of this review, global mass coral bleaching events since 1998 have occurred in 2010, 2014–2017, and most recently in 2023–2024. The National Oceanic and Atmospheric Administration (NOAA) confirmed this fourth global coral bleaching event was underway as of April 2024 (NOAA 2024). These events predominantly stem from prolonged marine heatwaves associated with El Niño occurrences, which cause extreme thermal stress on corals worldwide and trigger mass bleaching (Eakin et al. 2019; Oliver et al. 2021). Globally, marine heatwave duration and frequency have been increasing. Projections are indicating more rapid increases due to climate change (Oliver et al. 2018, 2021). With global temperature on the rise, and many other factors threatening corals globally and locally, coral bleaching rates have increased fivefold from 1980 to 2020 (Curran & Barnard 2021). To better understanding how coral reefs will be affected, researchers must continue to monitor coral reefs as well as model how projected climate change will alter these ecosystems. Bleaching will likely continue to increase if actions toward reducing greenhouse gas emissions, pollution, overexploitation, and other pressures on corals are not reduced.

## Mechanisms of bleaching

**Fig. 3:**
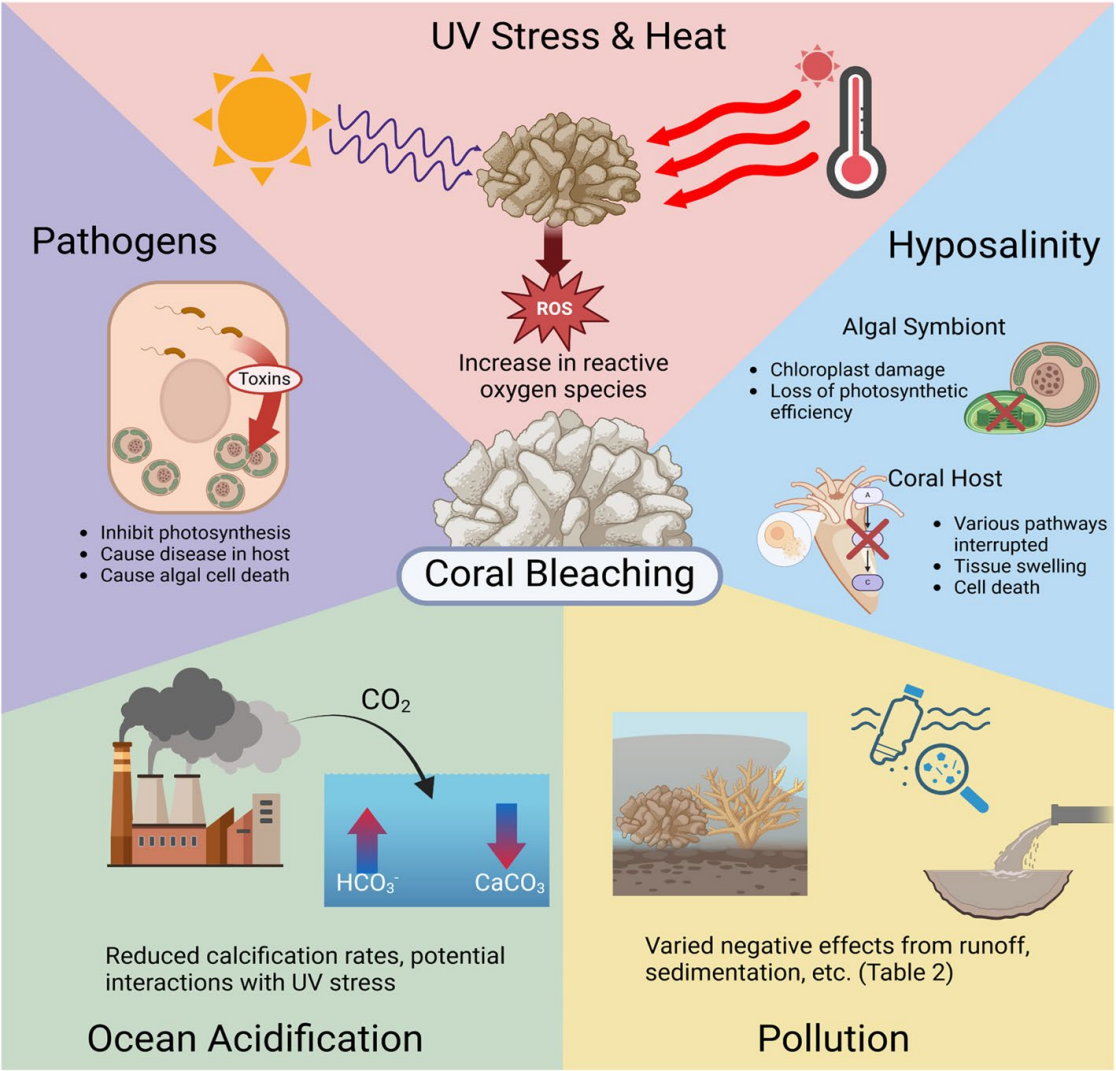
Summary of the commonly known causes of coral bleaching. More details on the mechanisms of each cause are provided in Table 1

The causes and mechanisms of coral bleaching (summarized in Table 1 and Fig. 3) are varied and complex; further research into the exact mechanisms and their relationships to each other is ongoing. Some common stressors that induce bleaching include thermal stress (Cleves et al. 2020), osmotic stress (Dias et al. 2019), pollution (Donovan et al. 2020; Haynes & Johnson 2000), ocean acidification (Silverman et al. 2009), excessive sunlight exposure (Brown 1997; Downs et al. 2013), pathogens (Kushmaro et al. 1996, 1997; Rosenberg & Falkovitz 2004; Rosenberg et al. 2009), and sedimentation (Duckworth et al. 2017). These stressors can exacerbate or moderate one another (Anthony et al. 2007; Bessell-Browne et al. 2017; Downs et al. 2013), as well as compound other threats to coral reef survival, such as changing corallivore populations (Cole et al. 2008; Renzi et al. 2022).

**Table 1:** Summary of Stressors and Mechanisms of Coral Bleaching

Stressor	Proposed Mechanism(s)	Source of Stressor
Heat and UV Radiation	Damage to photosynthetic machinery is associated with all light intensities. Photoinhibition occurs when such damage overwhelms the capacity for repair, such as with elevated temperatures or increased light intensity (Adir et al., 2003) Oxidative stress, which is associated with photoinhibition, induces damage to cellular components of both symbionts, initiating bleaching pathways (Blackstone 2022; Brown 1997; Cleves et al. 2020; Downs et al. 2013; Pei et al. 2022; Szabó et al. 2020; Tolleter et al. 2013; Weis 2008; Zhang 2022)	Climate change Seasonal variations El Niño-Southern Oscillation cycle
Salinity	Hyposalinity is correlated with host tissue swelling and necrosis, damage to and loss of zooxanthellae, reduced photosynthetic efficiency of zooxanthellae, accumulation of oxidative stress in both symbionts, and disruption to host detoxification and endocrine pathways (Downs et al. 2009b, 2013) Hypersalinity may improve thermal tolerance in coral hosts (Chen et al. 2023; Gegner et al. 2017; Randle et al. 2020)	Variations to global water cycle Environmental engineering
Pollution	A variety of pollutants cause varying effects ranging from photoinhibition of the zooxanthellae to necrosis of host cells. (Jones 1997, 2005; Jones et al. 2003; Markey et al. 2007). These pollutants are further explored in Table 2	Anthropogenic sources (i.e. waste water runoff, illegal dumping, etc.)
Acidification	Acidification has variable effects on the coral-zooxanthellae symbiosis For example, acidification may exacerbate bleaching caused by UV radiation (Anthony et al., 2008), whereas increased levels of bicarbonate may mitigate oxidative damage (Strohecker et al. 2020)	Climate change (Rise in CO_2_ emissions)
Pathogens	Certain pathogens may induce bleaching, such as the bacterium *Vibrio shiloi,* which infects the coral *Oculina patagonica*. *V. shiloi* produces Toxin P when exposed to temperatures of 25 °C-30°C, which can then cause photoinhibition (Banin et al. 2001a, 2001b; Kushmaro et al. 1996, 1997; Rosenberg & Falkovitz 2004)	Introduction of novel pathogens Alterations to reservoir/vector (Sussman et al. 2003)

These stressors induce physiological changes, including oxidative stress from the accumulation of ROS (Blackstone 2022; Szabó et al. 2020; Tolleter et al. 2013; Weis 2008), the inhibition of photosynthesis and the Calvin cycle in Symbiodiniaceae cells (Pei et al. 2022; Szabó et al. 2020), and abnormal host protein phosphorylation patterns (Sawyer & Muscatine 2001; Simona et al. 2019). If severe enough, these physiological changes may result in coral hosts removing algal cells via several mechanisms: activation of the host immune system, exocytosis, symbiophagy, host cell detachment, and apoptosis and/or necrosis (Bieri et al. 2016; Dunn et al. 2004; Gates et al. 1992; Mansfield et al. 2017; Oakley & Davy 2018; Pei et al. 2022; Sawyer & Muscatine 2001; Zhang et al. 2022). Symbiodiniaceae may also migrate to other coral tissue in order to avoid expulsion and repopulate the coral following a bleaching event (Parrin et al. 2016).

Exocytosis involves the expulsion of algal cells through the gastrovascular cavity and out of the host’s mouth. This process occurs on a normal basis as a means to control the algal population, but temperature- and light-related bleaching events may also trigger this response (Davy et al. 2012). Exocytosis-related bleaching occurs in response to a range of temperatures depending on factors such as coral species. The condition of Symbiodiniaceae cells being expelled may vary depending on temperature. Temperatures of 28℃ caused the exocytosis of viable algal cells, whereas temperatures higher than 28℃ resulted in a greater proportion of apoptotic/necrotic algal cells being expelled in scleractinian corals (Strychar et al. 2004). The exocytosis of photosynthetically active algal cells in *Cyphastrea sereilie* occurred at 33℃, with evidence of photoinhibition occurring beyond 37℃ (Ralph et al. 2001).

Symbiophagy involves the consumption of the algal symbiont within the host cell via phagocytic pathways. This mechanism is primarily correlated with thermal stress, as opposed to light stress (Downs et al. 2009a). The symbiosis between Symbiodiniaceae and the coral host is described as an arrested state of phagocytosis accomplished through the exclusion of the ApRab11 protein, which is associated with the recycling of endosomes and internalized transferrin (Davy et al. 2012; Chen et al. 2005). Oxidative stress can reactivate phagocytosis by interfering with proteins associated with the arrest of phagocytosis, leading to the in situ degradation of symbiont cells (Davy et al. 2012; Downs et al. 2009a).

Host cell detachment occurs in bleaching events relating to heat, cold, and chemical stressors (Bieri et al. 2016; Gates et al. 1992; Sawyer & Muscatine 2001). Detachment involves the dissociation of Symbiodiniaceae-containing coral cells, followed by expulsion using cilia and actinopharynx muscles. This process may be caused by disruptions to cell adhesion, such as membrane thermotropism, temperature-related denaturation of proteins, and changes in ion gradients (particularly that of calcium) that potentially interfere with cytoskeletal structures (Gates et al. 1992; Sandeman 2006). Another proposed explanation is that host cell detachment is not necessarily a mechanism of bleaching, but rather a result of extreme cellular stress (Buddemeier & Fautin 1993; Bieri et al. 2016).

Host cell apoptosis and/or necrosis may also occur as a method of bleaching (Dunn et al. 2004). Apoptosis is the process of controlled cell death, whereas necrosis is the process of uncontrolled/accidental cell death. Apoptosis provides an opportunity to avoid further cellular damage (e.g., the uncontrolled destruction of lysosomes) while allowing for the recycling of cellular components. The pathway of apoptosis and necrosis may be mediated by ROS, such as nitric oxide, serving as secondary messengers in the apoptosis/necrosis pathways (Laloi et al. 2004; Perez & Weis 2006). The source of ROS, either from the coral host or algal symbiont, is debated, but evidence shows that ROS can cross the symbiosome membrane (Szabó et al. 2020). Given this finding, additional research on the holobiont’s biochemical processes could illuminate the mechanisms underlying ROS production and export. Informing this knowledge gap may aid in finding means to mitigate ROS damage.

### Heat and light (UV)

Two of the most prominent causes of coral bleaching include high temperatures and UV radiation (Downs et al. 2002; Lesser 1997; Oakley & Davy 2018). Although usually grouped together, excessive heat and UV radiation can cause bleaching independently of each other (Oakley & Davy 2018; Tolleter et al. 2013). Temperature and light-associated bleaching involve the accumulation of oxidative stress, including superoxide radicals (O_2_^−^), singlet oxygen (1O_2_; not technically classified as a ROS but often associated with ROS production), hydroxyl radicals (OH), hydrogen peroxide (H_2_O_2_), and nitric oxide (NO) (Lesser 1996; Perez & Weis 2006; Szabó et al. 2020; Weis 2008). Within the algal symbiont, oxidative stress can accumulate in photosystem II (PSII) (Lesser 1996, 2011; Zavafer & Mancilla 2021), photosystem I (PSI) (Lima-Melo et al. 2021; Szabó et al. 2020), and the thylakoid membrane (Smith et al. 2005). These ROS can damage biomolecules, affect signal transduction, and affect cellular processes such as apoptosis (Bhatt et al. 2021; Edreva 2005; Lesser 2011). Under normal circumstances, the algal cell is able to degrade and replace damaged proteins (such as the D1 protein, a vital component in PSII) de novo (Kato & Sakamoto 2009). However, when oxidative damage overwhelms repair processes, the coral host cell may remove algal cells through the mechanisms discussed in the previous section.

Photoinhibition (the reduction of photosynthetic efficiency) is associated with thermal and light stress and is implicated in the bleaching process (Smith et al. 2005). Several mechanisms have been proposed to explain photoinhibition. These mechanisms are not necessarily exclusive and may work in conjunction to explain photoinhibition and associated oxidative damage (Zavafer & Mancilla 2021). For example, in response to high temperatures and irradiance, damage and reduction in D1 proteins of photosystem II and disruptions to the Calvin cycle have both been suggested as contributors to coral bleaching (Douglas 2003). The biochemical pathways of photoinhibition in symbiotic algae are a prominent gap in understanding how high irradiance may contribute to bleaching. In terms of physical mechanisms, the optical microhabitat (discussed earlier in this review) may play a role in exacerbating the effect of excess light. Due to the efficacy of the skeletal light-redistribution properties and the importance of host coral tissue in attenuating incident light, bleaching leads to far greater algal light exposure on bleached corals (Marcelino et al. 2013; Swain et al. 2018; Wangpraseurt et al. 2017). This effect creates a positive feedback loop where algal symbionts that survive initial bleaching events are at greater risk from photoinhibition, thereby worsening the bleaching. Research indicates that the optical feedback response differs by host morphology; tissue thickness contributes some resistance due to the presence of photoprotected microniches (Wangpraseurt et al. 2017). Additionally, corals with skeletal structures that are more efficient at redistributing light are predictably more at risk from the optical feedback loop (Marcelino et al. 2013). Further research in the optical microhabitat and potentially other physical mechanisms involved in irradiance would aid in understanding the contribution of light stress to coral bleaching and mortality.

### Salinity

Corals are osmoconformers, a group of organisms that maintain their internal fluids isotonic in relation to the environment (Aguilar et al. 2019). As such, they are susceptible to the osmolarity of the environment. Ocean salinity is influenced by the global water cycle, including seasonal changes (Durack et al. 2012; Stammer et al. 2021).

Low salinity is cited as another cause of coral bleaching, especially when combined with other factors such as high temperatures (Glynn 1991; Kerswell & Jones 2003; Kongjandtre et al. 2021). Despite its prevalence as a factor in coral bleaching, the exact mechanism by which salinity-associated bleaching could benefit from further study. Salinity-related bleaching likely involves damage to the chloroplast membrane (Downs et al. 2013), which is correlated with impairment of photosynthesis and subsequent expulsion (Jones et al. 1999; Kerswell & Jones 2003). Several other coral pathomorphologies are associated with hyposalinity, including host cell tissue swelling and death, loss of Symbiodiniaceae symbionts, decreased symbiont photosynthetic efficiency, disruption of detoxification and endocrine pathways, and increased oxidative stress in both symbionts (Downs et al. 2009b).

Conversely, other studies have found that high salinity conferred thermotolerance in corals and coral models such as *Aiptasia* spp. (Chen et al. 2023; Gegner et al. 2017; Randle et al. 2020). Although the exact mechanism of thermotolerance is poorly understood, it is hypothesized that floridoside plays a role. Floridoside is produced by both coral hosts and algal symbionts in response to high saline conditions. It has a dual role, acting both as an osmolyte and an antioxidant. The osmolytic property is what likely plays a role in mitigating the bleaching response (Ochsenkühn et al. 2022). Other potential explanations include the correlation between high salinity and general stress resilience (Chen et al. 2023; Gegner et al. 2017), as well as shifts in the coral microbiome, including bacteria implicated in nitrogen cycling (Randle et al. 2020).

Besides its mitigating effects on coral bleaching, high salinity is correlated with short-term impairment (and eventual acclimatization) of coral calcification and photosynthetic activity in Symbiodiniaceae. This impairment is likely facilitated by changes to the coral microbiome (Roethig et al. 2016). Once again, this highlights the need for a better understanding of the microbiome and how it influences holobiont resilience.

### Pollution

The wide variety of different pollutants and their respective effects necessitates that this review focuses on a select few pollutants associated with bleaching. The effects of pollutants on coral bleaching vary greatly depending on a number of factors, including exposure length, type of pollutant, coral species, coral life stage, and solubility of pollutant. Common routes of pollution include short-term acute events (e.g., oil spills) and chronic events (e.g., terrestrial runoff). Pollutants known to induce coral bleaching include certain metals, cyanide, agricultural herbicides and pesticides, antifouling agents, organochlorines, and oil hydrocarbons (van Dam et al. 2011). Table 2 outlines a list of common pollutants for each category.

**Table 2:** Common bleaching-related pollutants and their effects on the coral-zooxanthellae symbiosis

Pollutant Category	Common Routes of Pollution	Common Effects	Examples
Metals	Agricultural and mining industries Waste runoff	● Potential incorporation into the coral skeleton and tissues (Howard & Brown 1984; Brown et al. 1991) ● Uptake of metals in zooxanthellae and subsequent bleaching (Jones 1997) ● Inhibition of photosynthesis (Jones 2005) ● Death of coral host (Jones 1997)	Copper Zinc Arsenic Cadmium Mercury Lead Nickel Aluminum Manganese Iron Tin
Biocides	Agriculture Antifouling agents	● Inhibition of photosynthesis (Jones et al. 2003; Owen et al. 2003) ● Impairment of reproduction and larval metamorphosis (Markey et al. 2007)	Diuron Atrazine Irgarol 1051 Endosulfan Chlorpyrifos Profenofos Carbaryl permethrin
Sedimentation	Dredging Illegal fishing practices	● Restricted solute exchange and/or reduced light transmission, resulting in bleaching but not mortality (Duckworth et al. 2017) ● Decreased net productivity, growth, calcification, and death of coral (Rogers 1990) ● The sediment smothering of corals was highly based on coral morphology (Smothering was not observed in branching species), which resulted in bleaching and partial mortality (Jones et al. 2019) ● High sediment improved coral survivorship under high light/high-temperature conditions (Anthony et al. 2007)	
Eutrophication	Agriculture Sewage runoff	● Increased severity of bleaching in elevated nitrogen levels (Donovan et al. 2020) ● Elevated nutrient levels, when combined with overfishing, can cause increased competition between coral and macroalgae, disruptions to the coral microbiome, increased susceptibility to bleaching and disease, and mortality (Zaneveld et al., 2016)	Fertilizers Nitrogen-based: ammonium, nitrate, nitrite Phosphate
Microplastics		● Minor species-dependent effect on thermal tolerance (Reichert et al., 2021)	
Hydrocarbons	Oil spills Shipping	● Polycyclic aromatic hydrocarbons (Often found in crude oil) caused bleaching when in conjunction with UV radiation (Carmen GuzmáN Martínez et al., 2007) ● Crude oil reduces the fitness of zooxanthellae (Müller et al., 2021)	

### Ocean acidification

In addition to rising temperatures, ocean acidification from climate change presents another serious threat to marine ecosystems, particularly reef-building corals. Ocean acidification primarily occurs via the uptake of atmospheric CO_2_ into the ocean. This leads to the lowering of pH, increased levels of bicarbonate (HCO_3_^−^), and decreased saturation states of carbonate (CO_3_^2−^). The latter of these effects can affect concentrations of calcium carbonate (CaCO_3_) used in calcification (Leung et al. 2022). Further research is necessary to better understand the relationship between ocean acidification and bleaching. One proposed mechanism relating ocean acidification and bleaching suggests that a lack of CO_2_ available to the algal symbiont may reduce the consumption of ATP and NADPH produced from the photosynthetic electron transport chain. This blocks the electron transport chain, causing photoinhibition and accumulation of ROS (Wooldridge 2009).

The effects of ocean acidification on coral-algal symbiosis vary according to several factors such as temperature, light exposure, region, coral life stage/age, and coral/Symbiodiniceae species (Bove et al. 2020; Jiang et al. 2022; Kornder et al. 2018). Different experimental models may have contradicting results, further complicating the effects of acidification. For example, Albright (2018) found that ocean acidification reduced coral calcification rates due to the depletion of aragonite and carbonate ions, and Anthony et al. (2008) found that UV radiation in conjunction with acidification caused bleaching. In contrast, Bove et al. (2020) found that regular calcification rates were observed under both heat stress and acidification conditions. Additionally, increased levels of bicarbonate have been proposed to mitigate the damaging effects of ROS, yet increased bicarbonate concentrations may also act as a stressor in the absence of light and thermal stress (Strohecker et al., 2020).

While not a definitive cause of coral bleaching, the effects of acidification on the coral-algal holobiont and coral reef calcification should not be overlooked. Acidification hampers coral reef resiliency and recovery through impairment of reproduction, sequential life history stages, and calcification (Albright & Cooley 2019; Cornwall et al. 2021; Hoegh-Guldberg et al. 2007).

### Pathogenic infections

Pathogenic microorganisms are also correlated with coral bleaching, usually in conjunction with elevated temperatures and/or elevated light. Some examples include *Vibrio shiloi*-induced bleaching in the coral *Oculina patagonica*, *Vibrio coralliilyticus-*induced bleaching in the coral *Pocillopora damicornis*, four *Vibrio* spp. which cause Yellow blotch disease (a bleaching-related disease) in *Montastrea* spp., and *Vibrio fortis* which infects the corals *Seriatopora guttatus* and *Pocillopora damicornis* (Kushmaro et al. 1996, 1997; Rosenberg & Falkovitz 2004; Sun et al. 2023).

The mechanisms by which pathogens induce coral bleaching vary. Consider the example of *V*. *shiloi,* which induces bleaching in the coral *O*. *patagonica* found in the Mediterranean Sea. *V*. *shiloi* adheres to a receptor present only in coral with a photosynthetically active endosymbiont population. At elevated temperatures of 25 °C-30°C, *V. shiloi* produces toxins that inhibit photosynthesis and cause bleaching/lysis of algal cells. One of these toxins, referred to as Toxin P, binds to algal cell membranes and creates a channel that enables the transport of NH_3_, but not NH_4_^+^. This affects the pH gradient of the thylakoid membrane and thereby inhibits photosynthesis, potentially triggering bleaching (Banin et al. 2001a, 2001b; Rosenberg & Falkovitz 2004). *V. shiloi* also produces the virulence factor superoxide dismutase (SOD) at elevated temperatures of 30 °C, which serves to protect *V. shiloi* from oxidative stress caused by heightened photosynthetic activity (Banin et al. 2003; Rosenberg et al. 2009). *V. shiloi* activity depends greatly on temperature; they are active in the summer when temperatures in the Mediterranean Sea range from 25–30 °C, but not during winter when temperatures drop to 16–20 °C. As such, rising global temperatures may induce pathogen-related bleaching events even in thermotolerant corals (Sussman et al. 2003). *V. shiloi* cannot survive in *O. patagonica* tissues in winter temperatures. Additionally, *O. patagonica* seems to have developed a degree of resistance to *V. shiloi* sometime between 2002 and 2004, as evidenced by the inability to detect *V. shiloi* in healthy and bleached corals (Reshef et al. 2006; Rosenberg et al. 2009).

## Ongoing strategies to address coral loss

As anthropogenic global climate change shows no signs of slowing, conservation efforts must adopt a multi-faceted approach toward mitigating the losses of corals and building a more resilient ecosystem. Strategies adopted to combat coral loss can be broadly described in two categories: restoration and assisted evolution. Restoration practices focus on mitigating and repairing damages to coral ecosystems while preserving key species of corals. For simplicity in this review, environmental interventions, coral transplantation/gardening, and larval propagation fall under restoration strategies. Assisted evolution addresses the concern of a permanently changed ocean environment and seeks to create a coral reef ecosystem that can withstand increasingly adverse conditions. This section primarily discusses selective breeding and assisted gene flow, but emerging frontiers in biotechnology are also explored. Several methods employ a combination of restoration and assisted evolution to revitalize and improve the overall health of coral reefs. However, coral loss intervention strategies vary greatly; many more examples exist within and beyond those discussed here.

### Coral restoration: establishing a practical toolkit

Restoration projects focus on helping native corals establish and maintain colonies. As preserving reef ecosystems is a pressing issue in many coastal communities, a wide variety of coral restoration programs have been put in place across the globe. These practices utilize a variety of methods spanning from a whole-environment approach down to the coral larvae and gametes. Studies in this sect of coral research aim to inform ongoing and future restoration programs on how to best improve coral survivorship while reducing costs.

#### Environmental intervention

The dire need for action regarding coral loss cannot be understated. In this respect, environmental intervention strategies may be the most convenient tool available to quickly deploy. Several types of intervention have been proposed and, of these, some are already being implemented; a select few discussed here illustrate the potential benefits of this kind of restoration work.

One of the simplest ways to help corals recover is to improve their degraded habitats. The disappearance of coral-dominated benthos often accompanies a phase shift to macroalgae-dominated benthos as various anthropogenic factors allow the macroalgae to outcompete corals (Ceccarelli et al. 2020; Hughes 1994; Jackson et al. 2014; Randazzo-Eisemann et al. 2021). In addition to competing for space, macroalgae may exert some stress on coral gametes and prevent the settlement of new corals (Cetz-Navarro et al. 2015). Management strategies for macroalgal removal have, therefore, proved an effective tool for promoting coral growth (Tanner 1995) and even improving the recruitment of new corals (Smith et al. 2022; Tanner 1995). While working toward the ultimate goal of mitigating anthropogenic factors, long-term implementations for macroalgae control should be investigated. A promising strategy in this type of intervention is a form of biocontrol whereby native grazers such as urchins are added to reefs to consume excess algae (Neilson et al. 2018). Strategies of this nature can be quickly implemented for results (NOAA 2017).

A restoration tactic that has long been favored is the use of artificial substrate for reefs. Artificial reefs have been well documented in the scientific literature for the past half century, but their use may date back far longer than that (Schuhmacher, 1977; Stone et al. 1979. See Hylekema et al., 2021 and Lee et al. 2018 for older examples). Historically, wrecks and tires were considered good artificial reefs for their structural complexity, but this is with minimal regard for the corals themselves. Tire piles and wrecks were easy to implement and had positive effects on species abundance, largely with respect to fish (although coral recruitment was seen, particularly on metal surfaces) (Arena et al. 2007; Collins et al. 2002; Fowler and Booth 2012). Additional studies have demonstrated the success of artificial substrate for coral settlement (Burt et al. 2009; Clark and Edwards 1994). Much research herein has been focused on testing different materials for coral recruitment. Concrete, gabbro (a type of coarse-grained igneous rock), and terra cotta have been identified as good substrates to date (Al-Horani and Khalaf 2013; Burt et al. 2009; Fitzhardinge and Bailey-Brock 1989; Lam 2003). With the effective materials identified and deployed, long-term studies for coral survivorship and overall ecosystem effects should be investigated. Implementation of artificial reefs is documented as far back as the 1970s in some instances, providing access to peer-reviewed long-term data (Schuhmacher, 1977; Stone et al. 1979). Additional research comparing artificial to natural reefs across time is needed to illuminate the effects of artificial substrate on corals in situ and the resulting changes to ecosystems as a whole.

The potential harmful effects of artificial substrate are largely understudied. A 2021 meta-analysis of Caribbean artificial reefs found that the benthic community of the synthetic substrate differed from natural reefs, in some cases including more non-indigenous species. The study also highlighted the potential for artificial substrates to introduce harmful chemicals and excess nutrients, such as iron, into the ecosystem (Hylkema et al. 2021). This is especially true in the case of tires which leach fouling agents into the water (Aleksandrov et al. 2002; Collins et al. 2002). Other long-term analyses of this nature further illustrate the unforeseen effects of certain types of artificial reefs on community assemblage (Monchanin et al. 2021).

Another sect of artificial reefs includes biomimetic structures, designed to mimic natural reef structures (Evans et al., 2021; Lange et al. 2020; Maslov et al. 2024). As discussed in the introduction, much of the diversity of coral reefs is due to great degrees of structural complexity. With the loss of coral and the threat of algae phase-shifts, biomimetic reefs may be helpful in mitigating biodiversity loss by mimicking this complexity (see Levy et al. 2022 for more information). In a further evolution of this field, researchers are also experimenting with microenvironments that could replicate coral-algal symbiosis. Although this research has yet to be scaled up, 3D bioprinting can be used to create coral polyp structures that can successfully host algal symbionts, thereby replicating to some degree the microhabitat of the coral reef (Wangpreseurt et al., 2022). On the whole, biomimetic environmental engineering could aid in preventing ecosystem collapse by preserving biodiversity while coral restoration strategies catch up. Additionally, promoting healthier reefs may have a positive effect on coral recruitment, leading to the natural restoration of reefs (coral recruitment is further discussed below). Much of this research is still in the design phases; more research is needed to evaluate the efficacy of biomimetic reefs. However, interest in this field marks an important transition to more ecologically informed solutions to reef restoration.

All strategies require long-term monitoring for proper implementation, but it is particularly relevant in environmental intervention strategies as there is an inherent risk in manufacturing ecological change (Hylkema et al. 2021). Great care should be taken to ensure that implementation is 1.) effective and efficient and 2.) does not lead to further unforeseen issues. While this review focuses mainly on the coral-based research available, it’s possible that other coastal management strategies in key locations have an unexpected effect on coral reef ecology. Long-term monitoring of such projects and their resulting influence on corals should also be considered in future studies.

#### Coral transplantation and coral gardening

A more direct approach to coral restoration is transplantation, often used in conjunction with a concept known as coral gardening. Coral gardening involves raising corals in protected nurseries before transplanting them into degraded reefs (Rinkevich 1995). Both asexual and sexual recruits can be used in this strategy, but neither should be the sole focus of restoration efforts. The two types complement each other and both are necessary for the most effective implementation. Asexual recruits farmed from fragments have demonstrated the success of coral gardening in a variety of protected in situ “nurseries'' (Dehnert et al. 2021; Guest et al. 2014; Shafir and Rinkevich 2010). Coral colonies grown this way may benefit from the relative cost and speed of implementation but lack genetic diversity (Levy et al. 2010; Mbije et al. 2013). To address this drawback, experiments on sexual recruits have also shown relative success (Chamberland et al. 2015; Guest et al. 2014; Miller et al. 2022; Pollock et al. 2017). Corals raised from larvae or gametes preserve genetic diversity, enhancing the overall population fitness (e.g., Allentoft and O’Brien 2010; Markert et al. 2010). However, there are greater growth times and costs necessary to rear corals in this way (dela Cruz and Harrison 2017; Guest et al. 2014). Additionally, transplantation success depends on the age and size of coral fragments reared from larvae. Young corals that spend longer in the nursery and are larger at the time of transplantation have significantly lower mortality rates (Guest et al. 2014). Shorter nursery times may not provide as much of a benefit beyond protected growth, particularly for slow-growing coral species (dela Cruz et al. 2015). In both sexual and asexual recruits, protected nurseries serve as efficient staging areas for coral transplantation, given their effectiveness in augmenting coral growth. Successful coral nursery operations can be scaled up to lower costs and improve output efficiency. With ever-improving methods, the coral gardening approach provides a cost-effective and robust strategy to restore degraded reefs (Rinkevich 2014).

Corals need not necessarily be reared in nurseries or “gardens” to be transplanted, however. Colonies existing in the environment may be relocated to improve their survivability. Rodgers et al. (2017) found that relocation to wave-protected areas from dredged sites allows corals to recover and flourish. This also establishes new reefs near the degraded ones in just a few years, thereby restoring the local ecosystem. Advantages of direct transplantation include lower costs and no rearing times. These benefits may come at the cost of decreased survivorship, as the direct comparison shows that fragmented corals are more likely to survive transplantations when grown in a coral nursery (dela Cruz et al. 2015). For a short-term span of several years, transplantation projects have been very successful and encouraging in their findings on coral fitness (Barott, 2021; Rodgers et al. 2017). However, they highlight the need for additional long-term monitoring of transplanted reefs in terms of longevity, resilience to stochastic events such as heat waves, and overall ecosystem effects. Future long-term research would benefit greatly from a standardized scoring system such as the one developed by Suggett et al. (2019) to evaluate the success of various transplantation strategies in the Great Barrier Reef.

A related branch of research, not discussed in detail here, is polyp bailout. Walton et al. (2024) show that coral polyps can be induced with manipulations to salinity and substrate to settle and grow into healthy micropropagates. These micropropagates can then be grown to transplant into reefs. The mechanics of polyp bailout are outside the scope of this review, but it is highlighted here for its relevance to coral restoration. Polyp bailout is an understudied tactic in coral propagation that would benefit from further investigation. For greater detail on this phenomenon and its applications, refer to Schweisenberg et al., 2021.

#### Larval propagation and settlement enhancement

Another avenue for restoration tactics focuses on the coral in their larval stage. As this review does not discuss the complexities of coral reproduction, the research considered in this section will focus on enhancing the survival and settlement of larval corals. The findings herein can inform future research and restoration practices for greater success in rearing coral from larvae.

Collecting coral gamete bundles as well as swimming larvae allows researchers to increase the amount of coral larvae available for settlement (dela Cruz and Harrison 2020; Linden and Rinkevich 2011; Suzuki et al. 2020). Artificially enhanced larval supply directly onto degraded reefs showed greater degrees of coral settlement and survivorship after 3 years when compared to natural plots (dela Cruz and Harrison 2017). Although it may be tempting to add a high density of coral larvae to improve settlement, studies have found that optimized densities improve both larval survivorship (Pollock et al. 2017) and settlement rates (Cameron & Harrison 2020). However, these findings may differ between species as Doropoulos et al. (2017) found larval survival was independent of density in *Acropora millepora*. Furthermore, the long-term survival of the young corals from settlement onwards is dependent on how crowded the substrate is. More densely settled substrates are associated with lower survivorship, likely because of increased competition (Suzuki et al. 2012). Therefore, coral larvae density needs to be optimized in practice using the best available knowledge for the target species.

Many studies on coral larvae are concerned with improving settlement on a variety of substrates. Larvae will preferentially settle in protected nooks such as small holes or crevices, presumably as protection from grazers (Doropoulos et al. 2017; Nozawa 2008; Okamoto et al. 2005). Additionally, crustose coralline algae (CCA) has long been identified as a preferred settlement site for competent larvae (Abdul Wahab et al. 2023; Diaz-Pulido et al. 2010; Heyward and Negri 1999; Morse et al. 1988). Studies built on findings like these can shed further light on the cues associated with larval settlement. For example, Foster and Gilmour (2016) found that red-colored tiles were preferentially settled when CCA sites were occupied, indicating that the red color of CCA may be a cue in the absence of other chemical cues. With further research on larval behavior, researchers can produce increasingly effective artificial substrates and enhance total coral larvae settlement. Though larval research is a promising tactic for increasing coral recruitment, there are many gaps in knowledge within the field. Miller et al. (2022) found that while settlement was feasible for in situ mesocosms, there were some occluding factors they could not identify that led to some trials seeing no settlement. Factors such as UV radiation (Aranda et al. 2011), temperature (Edmunds et al. 2001), and even sound (Vermeij et al. 2010) influence the behavior of larval corals. Acoustic enrichment, in particular, shows promise as a restoration tactic. Boulais et al. (2023) demonstrate that fish larval recruitment is 23 times higher when healthy reef sounds are played; these early findings indicate similar positive effects on coral larvae. The hidden complexities driving larval settlement are a gap in knowledge that could help advance the practical applications of larval research.

#### Interconnections of restoration strategies

The subcategories of coral restoration are not mutually exclusive; the findings within each category can be applied elsewhere for greater overall success. Coral gardening and larval propagation exist as independent strategies, but there is a distinct synergy between them. The relative success of sexually reared coral colonies is discussed in the coral gardening section; research that promotes larval survival and settlement allows for greater numbers of coral to be produced in this way, thereby increasing the overall nursery efficiency (dela Cruz and Harrison, 2020; Suzuki et al. 2020; Pollock et al. 2017). For in situ nurseries, there was an initially unexpected benefit of creating protected breeding populations capable of supplying the nearby environment with viable larvae (Amar and Rinkevich 2007; Shafir and Rinkevich 2010). Additionally, outplanted nursery corals contributed more to the larval pool than their native counterparts, perhaps due in part to increased fitness at the time of maturity (Horoszowski-Fridman et al. 2011). Long-term monitoring shows that this increased larval supply persists for several years after transplantation, indicating the presence of other hidden benefits from the nursery phase (Horoszowski-Fridman et al. 2020). While there is a gap in the literature about what could cause this observation, it highlights yet another synergistic interaction between coral nurseries and larval propagation.

There is also the possibility of using larval research applications in environmental intervention. For example, larval research often occurs on artificial substrates out of necessity, but the findings of these studies provide insight into the best practices for enhancing the natural environment. Artificial substrate additions should consider what kinds of properties, such as the red color Foster and Gilmore (2016) identified, induce greater degrees of coral larvae settlement. Supplying greater amounts of larvae onto degraded reefs has already proved successful, as discussed in the previous section (dela Cruz and Harrison, 2017). Coral larvae could likely be manipulated to settle in degraded areas without the need for artificial larvae supply as well. In algae-dominated environments, providing appropriate preferred substrates like highly rugose blocks (e.g., Nozawa 2008) could allow natural settlement and growth to occur where it previously did not. Additionally, further research on the behavior of swimming larvae could aid natural recruitment. Coral larvae can be induced to swim toward certain environmental cues, such as healthy reef sounds (Boulais et al. 2023; Vermeij et al. 2010). From both reef sounds and preferrable settlement areas, increasingly biomimetic artificial reefs could also aid the recruitment of coral larvae. In degraded environments near spawning populations, these kinds of cues can be implemented to passively influence the natural recovery of reefs.

### Assisted evolution: engineering the seeds of change

Reefs as a whole are able to independently evolve tolerance to adverse conditions, with some predicted as likely to survive even in warmer global temperatures. However, many more will be lost as temperatures rise (Matz et al. 2020). With strategies for restoration and management ongoing, it is important to look towards the future of coral conservation. More innovative coral research is in the early stages with the ultimate goal of understanding and manipulating the coral holobiont. Topics such as selective breeding, assisted gene flow, and gene editing fall under this umbrella of assisted evolution. Much of the research in this category has been with regard to projected temperature increases, although it is important to note that bleaching and subsequent coral death have many causes outside of temperature (as discussed in this review). Due to the lack of research in assisted evolution on other stressors, this section will specifically detail experiments conducted to enhance thermal tolerance.

#### Selective breeding

Heat-tolerant coral populations are being identified in many reefs worldwide (Lachs et al. 2023). Although the precise mechanisms for this observation need better characterization, it is clear that a combination of adaptation and acclimatization has allowed these corals to independently express heat tolerance (Palumbi et al. 2014). However, further intervention is needed to ensure the survival of reef ecosystems. One such intervention, selective breeding, identifies and produces heat-tolerant corals at a greater rate than natural selection. Selective breeding expedites the creation of thermally tolerant, stable coral populations.

In a recent study, researchers selected wild coral colonies to sexually propagate based on their thermal tolerance. An experimental warming period was used to check the potential parent colonies for heat tolerance, with a range of individuals’ gametes crossbred and reared from larvae (Humanes et al. 2021). As this kind of research is in its early stages, this study serves as a proof-of-concept for the utility of selective breeding as well as the challenges to anticipate in the future. In a further proof of principle, Humanes et al. (2024) show that adult coral heat tolerance is heritable. However, they find that short-term heat tolerance traits do not necessarily translate to long-term tolerance (Humanes et al. 2024). This indicates the need for both long-term studies that model marine heatwaves as well as a better understanding of the genetic mechanisms underlying thermal tolerance. Although true selective breeding would require long-term experimental conditions and many generations of corals, early results on selecting for thermal tolerance are promising. Barrot et al. (2021) found that transplanted corals that were selected for their bleaching resistance did not lose fitness when relocated and maintained their response to heat stress. This again demonstrates the feasibility of selective breeding in coral conservation.

Phenotypic plasticity (and, therefore, the likelihood of surviving in high temperatures) comes from a range of contributing factors in corals. In at least one species (*Montipora* *capitata*), genetics did not account for much of the phenotypic difference between bleaching and non-bleaching corals; the researchers indicated that symbiotic relationships play a greater role in holobiont fitness (Drury et al. 2022a, 2022b). Furthermore, one study found that different symbionts were associated with up to 14% of differentiated gene expression in the host, suggesting that symbionts are directly involved in host gene regulation (Cunning and Baker 2020). The particular interactions of host genetics and symbiont on phenotypic plasticity also merit further investigation to elucidate the factors responsible for heat stress responses. Some research has already been completed in this respect: Quigley et al. (2020b) tested crossbred juvenile coral with three different Symbiodiniceae species to determine how host genetics and associated symbionts contribute to thermal tolerance. The findings indicated that while symbiont species explained most of the bleaching response, certain genotypes from warmer reefs had greater comparative growth and survival rates (Quigley et al. 2020b). Further research on the interactions requires a better understanding of both host and symbiont genetic expression.

To this end, Symbiodiniceae projects are underway in parallel with the holobiont selective breeding research. In one study, researchers successfully raised the thermal tolerance of Symbiodiniceae over the course of 2.5 years. The selectively bred cells showed greater photosynthetic activity and had less extracellular ROS than wild-type cells at 31 °C. However, the cells’ increased thermal tolerance provided no significant benefits when introduced to growing coral fragments (Chakravarti et al. 2017). A similar study with 4 years of selective breeding on the symbionts corroborates the in vitro increase in thermal tolerance as well as a lack of consistently proportional benefit when inoculated to coral hosts. However, three of the heat-evolved strains (out of a possible 10) conferred thermal tolerance to their hosts; gene expression analysis on both host and symbiont identified highly differentiated expression for both (Buerger et al. 2020). The findings of these two studies indicate the feasibility of selectively breeding Symbiodiniceae for thermal tolerance, but again highlight the need for a better understanding of the factors influencing the complex symbiotic relationship.

#### Assisted gene flow

There is no guarantee that locally adapted benefits in coral populations can confer fitness to other populations. In fact, it would take far too long for these “well-adapted” individuals to spread to distant reefs (Quigley et al. 2019). In the meantime, loss of genetic diversity will afflict coral populations regardless of fitness (source if can find). Researchers have attempted to combat these impairments by facilitating the interbreeding of populations that would be unlikely to breed otherwise. Termed assisted gene flow (AGF), this manipulation is one way to prepare coral reef ecosystems for less hospitable conditions.

By crossing individuals from populations with different microclimates, researchers were able to produce a variety of traits representing a spectrum of coral fitness. Although purebred crosses had higher survivorship, some hybrids had other traits (such as decreased time to reproductive maturity) that demonstrated fitness in their new environments (Quigley et al. 2021). AGF can thereby augment local coral populations with traits from distant populations. To this end, selective breeding can also successfully increase the genetic variation of coral populations (Quigley et al. 2020a). This improves the likelihood that some individuals have traits to adapt to changing environments, further benefiting coral population survival.

One further possibility for AGF is the interspecific hybridization of two closely related coral species. Evidence suggests that *Acropora* hybrids could reproductively persist in the wild (Isomura et al. 2016). Furthermore, Banaszak et al. (2018) achieved interspecific hybridization with *Acropora* corals and showed that the hybrid offspring did not suffer from a loss of fitness. The specific value of this kind of hybridization has yet to be understood, but it is clear that this is a feasible way to increase genetic diversity in coral populations. AGF by hybridization would benefit from more research in situ on fitness and long-term (multigenerational) monitoring of hybrid offspring.

#### Biotechnology innovations

Various other solutions seek to aid in engineering corals through means other than those listed above. This section seeks to highlight a few of the innovative projects that work outside of the boundaries of restoration and delve into more potentially powerful (though understudied and perhaps uncertain) means of protecting coral reefs. Although not all of the examples below fit the description of assisted evolution, they are categorized here because, like assisted evolution, the nature of these projects involves fundamentally changing the coral holobiont itself. The aim of this is similar to that of selective breeding or AGF: creating more rapidly deployed solutions to coral bleaching. Though many more projects exist under the broad umbrella of biotechnology, those selected here are chosen for their applicability and promising early results.

One potentiality for quickly conferring fitness is microbiome manipulation. As discussed previously, corals are closely linked to a host of microbes that affect their fitness. Although microbiome manipulation is as-yet underexplored, early findings show positive results towards both resistance and resilience to bleaching. When corals were inoculated with microorganisms that had been screened for beneficial effects, they were more resistant to bleaching (Rosado et al., 2018). Additionally, Santoro et al. (2021) found that artificially enhancing the coral microbiome with select beneficial microbes helps the host survive post-bleaching by downregulating genes involved in post-heat stress disorder. However, they were unable to detect the added microbes at the end of the 15-day recovery period, indicating dynamic interactions with the host during stress recovery. This again demonstrates the need for research in coral host-microbe interactions. Greater availability of such information would enhance the potential to create microbiomes that aid in coral tolerance. In addition, long-term studies are needed to understand how manipulating the microbiome affects both the coral and the environment. A potential strategy that has been understudied for coral algae is the experimental mutagenesis of Symbiodiniceae cells to produce more heat-resistant strains. Gornik et al. (2022) demonstrate that Symbiodiniceae species *Breviolum minutum* can be transformed with exogenous genes. This study serves as a proof-of-concept for further research on bioengineering Symbiodiniceae, particularly as genomes become better characterized from ongoing genetic research. An analogous research project on symbiotic algae in a freshwater hydra showed that experimentally mutated symbionts were able to confer UV-B resistance to their hosts (Ye et al. 2020). Although this is not directly related to coral, the findings of this study are promising in terms of the feasibility of this strategy for the coral holobiont.

Various other advancements in biotechnology seek to provide multifaceted solutions to coral preservation by targeting areas of need. Some research here seeks to create long-term cultures of single coral polyps or coral cell lines to simplify in vitro study and experimentation (Nowotny et al., 2021; Pang et al. 2020; Rosental et al. 2017; Shapiro et al. 2016). Other experiments provide more accurate methods of assessing in vivo cellular dynamics, such as O_2_ flux, using nanoparticle technology (Fabricius-Dyg et al., 2012; Koren et al., 2016; Kühl et al. 2024). These examples are more about the benefit of coral research on the whole rather than the immediate applicability of rescuing corals, but there are innovative projects focused on the bleaching threat as well. Nanoparticle treatments engineered to reduce excess ROS from heat stress can improve both coral and algal survivorship (Motone et al 2018; Rogers et al. 2022). In a combination of both strategies discussed in the previous two paragraphs, there is also the possibility of engineering a microbiome specifically evolved for thermal tolerance (Maire and Van Oppen, 2022). Much of biotechnology engineering for coral solutions is still in the early stages, but there are many potential applications of emerging research here (see Roger et al. 2023 for more information). Innovation and new frontiers in coral science are increasingly necessary as the bleaching threat worsens.

### Integration of the major strategies

Restoration and assisted evolution tactics are not mutually exclusive. The intention has always been to apply assisted evolution outcomes and restoration techniques in conjunction to promote the growth and preservation of coral reefs. As of right now, many of the assisted evolution strategies are in experimental phases. However, some studies have shown success in the integration of the two methods.

#### Integration of multiple approaches

One study shows that 8 months of coral acclimatization in the favorable conditions of a coral garden, while associated with some drops in thermal tolerance, still retained traits of heat resistance overall (Morikawa and Palumbi 2019). Selectively bred populations benefit from coral garden optimization and can be conveniently reared in these kinds of nurseries for future outplanting. Humanes et al. (2021) showed that a longer nursery phase for selectively crossbred corals conferred various benefits to fitness, improving the likelihood of the young corals surviving outplanting. With the relative success of selectively bred sexual recruits, future research can benefit immensely from improvements to larval propagation methods as well as coral gardening advancements. Additionally, evidence suggests that transplanted corals show no loss of heat-adaptive traits when relocated (Barott et al. 2021). It is important to preserve genetic diversity in assisted evolution strategies to prevent bottlenecking and reducing the fitness of coral populations as well; coral nurseries were unexpectedly able to help with this by being somewhat protected. During one stochastic cold-water event, many coral colonies that were killed in the reef had surviving fragments in a pre-established nursery, thereby preserving those genotypes and the genetic diversity in populations (Schopmeyer et al. 2012). For the AGF branch of assisted evolution to be effective, many diverse populations need to be available. Restorative protection of reefs ensures that those genetic nuances are not lost in the meantime.

**Fig. 4:**
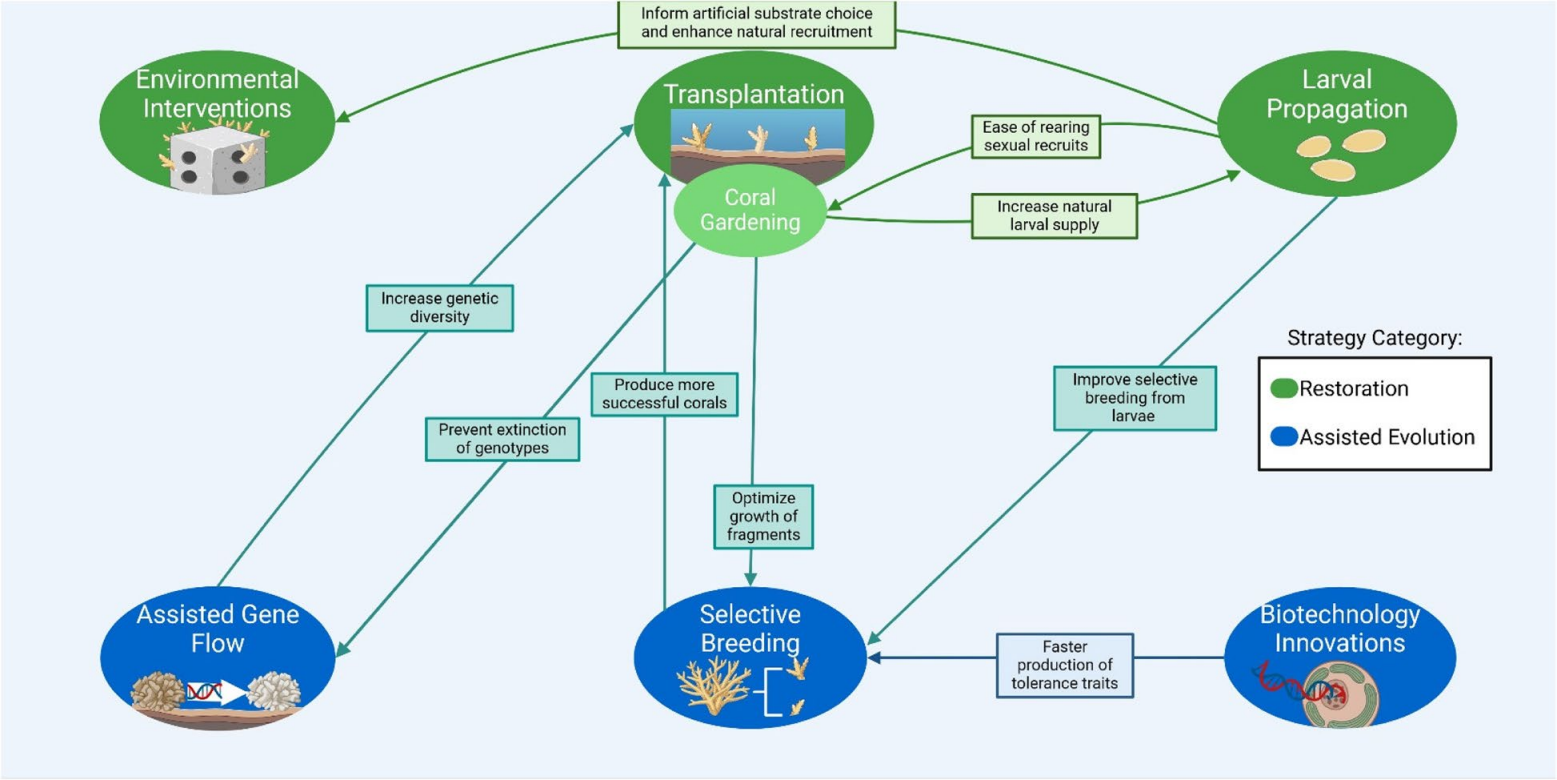
The web of strategies to address coral loss and the synergistic interactions between them. Strategies are characterized as either primarily restoration or assisted evolution. Benefits conferred from one strategy to another are indicated by an arrow in the direction of the benefit. Wide-scale benefits not depicted in the figure include the genetic characterization to inform restoration efforts from assisted evolution research and the availability of coral for experimentation supplied by restoration practices

Assisted evolution can reciprocate a benefit to restoration-based strategies as well: the data supplied by assisted evolution experiments can provide insight into restoration practices. A necessary step in the assisted evolution of the coral holobiont is genetic characterization; much of the genetic work being done on corals focuses on identifying factors responsible for heat tolerance so that the mechanisms behind it can be understood and manipulated. These data can inform outplanting efforts regarding the relative likelihood of survival for transplanted corals and thereby increase restoration efficiency (Drury and Lirman 2021; Fuller et al. 2020). Even if the widespread application of assisted evolution is not adopted, there are practical uses for this kind of research in restoration. The strategies and their interactions are summarized in Fig. 4.

#### Challenges of integration

As restoration methods are well-established and widely implemented, many encourage a focus on the expansion of these strategies for greater efficiency (e.g., Vardi et al. 2021). Conversely, advocates for the importance of assisted evolution point out that corals need to survive bleaching events in order to contribute to future reef ecosystems, a consequence that wholesale restoration does not address (Caruso et al. 2021). The critical necessity of ongoing restoration work should not be trivialized. However, experimental avenues of coral research need to be given proper attention as well. Assisted evolution may be in the early stages and largely not feasible for deployment, but some early adopters are already employing the strategies successfully (e.g., assisted gene flow; see Caruso et al. 2021 in conjunction with ‘How do We Restore Corals?’, n.d.). Long-term monitoring of these strategies will be especially important to evaluate both the success of implementation and the potential drawbacks. A common drawback (and therefore criticism) in selective breeding is the trade-offs that occur when selecting for heat tolerance. For example, corals with higher thermal tolerance tend to suffer lower growth rates as a result (e.g., Cornwell et al. 2021; Jones and Berkelmans 2011; Ladd et al. 2017; Matsuda et al. 2023). And yet improving genetic diversity within native populations potentially mitigates trade-offs–this is an instance where AGF would be beneficial.

It is worth noting as well that human interventions like assisted evolution have ethical concerns attached to them (Filbee-Dexter and Smajdor 2019). Although these concerns merit discussion, the ethical implications of artificial selection are beyond the scope of this review. With due consideration and awareness, advances in coral-assisted evolution can greatly improve the capacity for restoration while being proactive about climate concerns.

## Conclusions

In either gradual temperature rise (such as in anthropogenic climate change), or sporadic temperature fluctuations (such as those caused by the El Niño and La Niña climate patterns), coral bleaching is an imminent and looming threat. Coral biology encompasses fields ranging from molecular mechanisms of photoinhibition and oxidative damage, to the complex interactions of coral hosts, algal symbionts, and other associated microorganisms. Given the complexity of the coral-Symbiodiniaceae symbiosis, and the coral holobiont in general, coral bleaching likewise involves diverse stressors and mechanisms. Understanding these mechanisms is of paramount importance in order to best implement coral conservation, restoration, and related bioengineering practices.

Coral reefs, along with their numerous benefits, are at great risk from anthropogenic climate change. This review is meant to serve as an overview of the work done concerning corals and their importance in the face of climate change. While not exhaustive, the information cited here elucidates the variety of branching fields in coral research and highlights areas of limited understanding. Investigation into the mechanisms of coral bleaching and their various causes continues even as new strategies for management are proposed. However, even with ever-greater insights on coral holobiont biology, no amount of well-informed management strategies can mitigate the need for decisive climate change action.

## Data Availability

All data generated or analyzed during this study are included in this published article.
